# The effect of passive leg raising test on intracranial pressure and cerebral autoregulation in brain injured patients: a physiological observational study

**DOI:** 10.1186/s13054-023-04785-z

**Published:** 2024-01-16

**Authors:** Antonio Messina, Agnieszka Uryga, Alberto Giardina, Pietro Ciliberti, Denise Battaglini, Nicolo’ Patroniti, Marek Czosnyka, Xavier Monnet, Maurizio Cecconi, Chiara Robba

**Affiliations:** 1 IRCCS Humanitas Research Hospital, Via Manzoni 56, 20089 Rozzano, Milan, Italy; 2 Department of Biomedical Sciences, Humanitas University, via Levi Montalcini 4, Pieve Emanuele, Milan, Italy; 3 Department of Biomedical Engineering, Faculty of Fundamental Problems of Technology, Wroclaw University of Science and Technology, Wrocław, Poland; 4 Department of Surgical Sciences and Integrated Sciences, University of Genoa, Genoa, Italy; 5 Anaesthesia and Intensive Care, San Martino Policlinico Hospital, IRCCS for Oncology and Neuroscience, Genoa, Italy; 6 Brain Physics Laboratory, Addenbrooke’s Hospital, Cambridge, UK; 7 AP-HP, Service de Médecine Intensive-Réanimation, Hôpital de Bicêtre, DMU 4 CORREVE, Inserm UMR S_999, FHU SEPSIS, CARMAS, Université Paris-Saclay, 78 Rue du Général Leclerc, 94270 Le Kremlin-Bicêtre, France

**Keywords:** Neuromonitoring, Intracranial pressure, Passive leg raising, Fluid therapy, Hemodynamic monitoring

## Abstract

**Background:**

The use of the passive leg raising (PLR) is limited in acute brain injury (ABI) patients with increased intracranial pressure (ICP) since the postural change of the head may impact on ICP and cerebral autoregulation. However, the PLR use may prevent a positive daily fluid balance, which had been recently associated to worse neurological outcomes. We therefore studied early and delayed effects of PLR on the cerebral autoregulation of patients recovering from ABI.

**Materials and methods:**

This is a Prospective, observational, single-center study conducted in critically ill patients admitted with stable ABI and receiving invasive ICP monitoring, multimodal neuromonitoring and continuous hemodynamic monitoring. The fluid challenge consisted of 500 mL of crystalloid over 10 min; fluid responsiveness was defined as cardiac index increase ≥ 10%. Comparisons between different variables at baseline and after PLR were made by paired Wilcoxon signed-rank test. The correlation coefficients between hemodynamic and neuromonitoring variables were assessed using Spearman’s rank test.

**Results:**

We studied 23 patients [12 patients (52.2%) were fluid responders]. The PLR significantly increased ICP [from 13.7 (8.3–16.4) to 15.4 (12.0–19.2) mmHg; *p* < 0.001], cerebral perfusion pressure (CPP) [from 51.1 (47.4–55.6) to 56.4 (49.6–61.5) mmHg; *p* < 0.001] and the pressure reactivity index (PRx) [from 0.12 (0.01–0.24) to 0.43 (0.34–0.46) mmHg; *p* < 0.001]. Regarding Near Infrared Spectroscopy (NIRS)-derived parameters, PLR significantly increased the arterial component of regional cerebral oxygen saturation (O_2_Hbi) [from 1.8 (0.8–3.7) to 4.3 (2.5–5.6) μM cm; *p* < 0.001], the deoxygenated hemoglobin (HHbi) [from 1.6 (0.2–2.9) to 2.7 (1.4–4.0) μM cm; *p* = 0.007] and total hemoglobin (cHbi) [from 3.6 (1.9–5.3) to 7.8 (5.2–10.3): *p* < 0.001]. In all the patients who had altered autoregulation after PLR, these changes persisted ten minutes afterwards. After the PLR, we observed a significant correlation between MAP and CPP and PRx.

**Conclusions:**

In ABI patient with stable ICP, PLR test increased ICP, but mostly within safety values and thresholds. Despite this, cerebral autoregulation was importantly impaired, and this persisted up to 10 min after the end of the maneuvre. Our results discourage the use of PLR test in ABI even when ICP is stable.

**Supplementary Information:**

The online version contains supplementary material available at 10.1186/s13054-023-04785-z.

## Introduction

Fluid infusion to correct hemodynamic instability is one of the commonest interventions in intensive care unit (ICU) [1–3], but predicting fluid responsiveness, i.e., increase in stroke volume (SV) or cardiac output (CO) after a fluid challenge (FC), is still challenging [3–5]. After early resuscitation, tailoring fluid therapy is a key component of both the optimization and stabilization phases [5–7], when the response to fluid administration is limited to roughly a half of ICU patients, and physicians should achieve an effective fluid resuscitation, and limit the risk of fluid overload [2, 5, 8].

Tailoring implies testing preload dependence and the passive leg raising (PLR) is the most reliable, studied and widespread functional hemodynamic test adopted in ICU to predict fluid responsiveness. It has been extensively investigated in different subgroups of critically ill patients [9, 10]. PLR is supposed to be potentially harmful in the acute phase of acute brain injury (ABI) with unstable intracranial hypertension, since the postural change of the head may increase cerebral blood flow and, in turn, the intracranial pressure (ICP) [10–12]. However, its use may be still valuable in the following stabilization phase of ABI for adequately titrating daily fluid balance.

Optimal volume management in acute brain injured patients is still controversial, shifting from dehydration therapy (aimed at limiting cerebral edema) some decades ago [13], toward normovolemia or even hypervolemia today [14]. In 2018, the European Society of Intensive Care Medicine (ESICM) consensus on fluid therapy in neurointensive care found no high-quality investigations on this topic, and consensus-based practice recommendations suggested that clinicians should aim for normovolemia, and avoid a negative fluid balance [15]. In addition, a recent survey highlighted the literature gap and the need for a deeper evaluation of this topic [16] while the prospective CENTER TBI study found that significant variability exists across countries in fluid management [17]. Interestingly, the prospective CENTER TBI study found that a mean positive daily fluid balance was associated with higher ICU mortality (odds ratio [OR] 1.10 [95% CI 1.07–1.12] per 0.1 L increase) and worse functional outcome (1.04 [1.02–1.05] per 0.1 L increase) [18]. Accordingly, titrating fluid therapy in the stabilization phase of ABI could be crucial and the PLR is still considered the most reliable functional hemodynamic test for predicting fluid responsiveness and avoiding fluid overload [16].

While adopting PLR to assess fluids responsiveness in the acute phase of ABI may be considered obviously too risky for the patient, its use in the delayed phase of this syndrome when ICP is no more actively treated and all the other physiological variables associated to ICP management are controlled has not been investigated.

We hypnotized that PLR could be considered safer in the delayed phase of ABI, as compared to the acute phase, with a reduced impact on ICP and cerebral perfusion.

Therefore, we conducted an observational study, with the primary aim to assess the effect of PLR on cerebral autoregulation, ICP and cerebral oxygenation of patients with ABI during the maneuver and after 10 min after the end of the test.

Secondary aims were to assess these changes according to fluid responsiveness (responders vs non responders) and to evaluate the correlation between systemic and cerebral variables before and after PLR test.

## Methods

This prospective, observational, study was conducted at the Neurocritical Care Unit of San Martino Policlinic Hospital, IRCCS for Oncology and Neurosciences, Genoa, Italy, from 1st February 2021 to 1st February 2023 and was approved by the local ethics review board (Comitato Etico Regione Liguria, protocol n. CER Liguria: 23/2020). This study was conducted according to the “Strengthening the Reporting of Observational Studies in Epidemiology (STROBE)” statement guidelines for observational cohort studies [19] (Additional file 1: Table S1).

Inclusion criteria were: (1) adult patients (> 18 years old), admitted to the ICU following ABI [Traumatic brain injury (TBI), subarachnoid hemorrhage (SAH) or intracranial hemorrhage (ICH)], who were intubated and mechanically ventilated and who received invasive ICP monitoring and other multimodal neuromonitoring tools including cerebral autoregulation (using Intensive Care Monitoring), ICM+ and noninvasive cerebral oxygenation (using Near Infrared Spectroscopy, NIRS). The decision to insert invasive ICP monitoring was made according to clinical needs and to the Guidelines for the management of ABI, in the case of GCS < 9 and a positive CT [20–22]; (2) use of a PLR before performing a FC in the absence of acute hemodynamic instability needing prompt resuscitation and of unstable ICP. As a safety measure and according to local protocol, PLR test was performed if the patient had ICP < 22 mmHg for at least 48 h prior the PLR start; (3) only need for tier 0 treatment management to current guidelines. According to current Guidelines, tier 0 treatments consist in basic measures for controlling ICP which include: intubation and mechanical ventilation, serial evaluations of neurological status, analgesia and sedation to achieve comfort, avoidance of fever [23].This suggests that patients were stable from an ICP perspective as they did not require any specific strategy for ICP control.

Exclusion criteria were the absence of informed consent and the occurrence of ICP > 22 mmHg in the previous 48 h or the need of tier > 0 for ICP control.

### Patients’ clinical management

Patients were managed according to local protocols and current Guidelines [20–22]. In the ICU, patients  were intubated and mechanically ventilated in pressure or volume-controlled ventilation and were sedated with propofol (3–6 mg/kg/h) and/or midazolam (0.03–0.2 mg/kg/h) and fentanyl (0.1–0.8 µg/kg/min). Mechanical ventilation was set using tidal volume of 6–8 mL/kg of predicted body weight (PBW), and eventually higher tidal volumes were applied but maintaining plateau pressure < 27 cmH_2_O and driving pressure < 15 cmH_2_O. Patients were put in a head­-elevated position at 30°. Intracranial hypertension was managed in a stepwise approach, according to the most recent Seattle algorithm [23].

The decision to perform a PLR test was based on clinician’s evaluation and, in particular, according to local protocol of hemodynamic management of ABI patients. It was triggered by suspected cerebral hypoperfusion (defined as cerebral perfusion pressure (CPP) < 60 mmHg) and at least one sign of systemic hypoperfusion (prolonged capillary refill time > 3 s or high mottling score or central venous oxygen saturation < 70% [2, 5, 24]) in order to assess fluid responsiveness before FC administration.

PLR test was performed, as previously reported, with a gradual change in the head position toward a 0° position and then elevation of the lower limbs to 45° [10]. This position was maintained for 1 min, according to previously published PLR investigations. In the case of increased ICP > 25 mmHg during the maneuver, PLR test was terminated, and the patient was immediately positioned in the head-elevated position at 30°. If increased ICP persisted for 5 min, a bolus of mannitol or hypertonic saline (at 5% concentration, 100 mL) was administered. FC was started after the PLR test was terminated.

Data on neuromonitoring and hemodynamic parameters were collected at baseline (before PLR test), when the changes induced by PLR were maximal (within 1 min, early effects [10]), and 10 min after the PLR test (late effects). We used the last arterial blood gas sampled before PLR test, and the first available after PLR test.

### Neuro and hemodynamic monitoring

ICP was continuously monitored using an intraparenchymal probe or an external ventricular drain, according to clinical indications and current local practice. Arterial blood pressure was monitored in the radial or femoral artery zeroed at the level of the right atrium (Baxter Health- care CA, USA; Sidcup, UK). Cerebral autoregulation was measured using the ICM + software, which is able to provide real-time integration of ICP analysis and cerebral perfusion pressure to obtain an autoregulatory index, calculating the pressure reactivity index (PRx) (ICM©, http://www.neurosurg.cam.ac.uk/icmplus Cambridge Enterprise, Cambridge, UK), which is calculated over a 5-min moving window as the Pearson correlation of 30 consecutive 10-s average values of ABP and ICP, as previously described [25, 26]. According to current literature, a preserved autoregulation was defined for values of PRx below 0.3.

Cerebral oxygenation was measured using non-invasive, continuous, regional cerebral oxygen measurement through ‘Masimo O3 regional Oximetry monitor’ (USA), using a bilateral sensor applied in the frontotemporal region. This tool allows the calculation of different variables, including: rSO_2_ (representing the total value of regional cerebral oxygen saturation); variation from baseline in the oxygenated Hb of the total rSO_2_, thus representing changes in the arterial component of rSO_2_ (ΔO_2_Hbi); variation in the deoxygenated component of Hb within the total calculation of rSO_2_, thus representing changes in the venous component of rSO_2_ (ΔHbi), and cHbi, which is the sum of the values of ΔO_2_Hbi and ΔHbi (https://www.masimo.it/technology/brain-monitoring/cerebral-oximetry).

The hemodynamic monitoring was obtained by connecting the arterial pressure to the MOSTCARE™ system (Vytech Health, Padua, Italy) as previously used in ICU patients [27–29]. The high-fidelity signal sampling allows the MOSTCARE™ device to accurately recognize the dicrotic notch (and the associated dicrotic pressure, *P*_dic_) that defines the systolic/diastolic component of arterial wave. The device calculates the systemic impedance by analyzing the profile of the ‘points of instability’ related to arterial mechanics and backflow waves [27, 28] on a beat-to-beat analysis and finally estimates stroke volume (SV) and cardiac index (CI) and measures systemic arterial pressure (i.e., systolic (SAP), mean (MAP) and diastolic (DAP) arterial pressures). Arterial elastance is calculated by MOSTCARE™ as the ratio between *P*_dic_ and SV. All the hemodynamic values were visualized on the screen and stored in the hardware. To ensure the quality and consistency of hemodynamic data, the pressure signal was checked on the screen and the adequacy of the  waveform was evaluated by a square-wave test before starting the study protocol [30].

The FC consisted of 500 mL of crystalloid solution infused over 10 min [4, 31] and the patient was considered a fluid responder when CI increased ≥ 10% after FC administration.

### Statistical analysis

The normality of the data was assessed using the Shapiro–Wilk test. Because of the lack of normality distribution for most of the analyzed parameters and limited observations, non–parametric tests were applied, and data are presented in the text and in the tables as median and interquartile range.

Comparisons between different variables at baseline and PLR were made by paired Wilcoxon signed-rank test. The difference between patients regarding changes in CI (10% threshold) was tested using U Mann–Whitney test. A rank-biserial correlation (rrb) coefficient was used as the metric of a size effect. The correlation coefficients (95% confidence interval (CI)) between systemic and the different neuromonitoring variables were assessed using Spearman’s rank test. The rectangles around the plot of the correlation matrix are based on the results of hierarchical clustering. The level of significance was set at 0.05 in all analyses.

Statistical analysis was performed using STATISTICA 13 (Tibco, Palo Alto, USA) and R Statistical Software (v.4.0.2; R Foundation for Statistical Computing, Vienna, Austria) using ‘ggstatsplot’ [10]. Data are presented as median (first–third quartile) unless indicated otherwise. No significant differences were found in systemic hemodynamic parameters.

## Results

During the study period, a total of 118 patients with ABI were admitted to ICU and were considered for inclusion; 65 patients were excluded as they did not undergo multimodal neuromonitoring or did not require for clinical reasons a PLR test during neuromonitoring. Finally, 30 patients were excluded because did not have a stable ICP > 48 h with tier 0 treatment, when PLR was needed.

A final number of 23 patients were included in the analysis. The characteristics of the patients are presented in Additional file in the Supplementary Materials 1: Table S2. The median age was 62 (43–67) years, and 56.5% were male; 10 patients (43.5%) were admitted for TBI, 8 (34.8%) for SAH, and 5 (21.7%) patients for ICH. Hypertension was the most common preinjury comorbidity (7 patients, 30.4%); median GCS at admission was 7 (3–11); 13 (56.5%) patients received an intraparenchymal bold and 10 (43.5%) an external ventricular drain for ICP monitoring. Twelve patients (52.2%) were fluid responders.

### Effects of PLR test on multimodal neuromonitoring

**Fig. 1:**
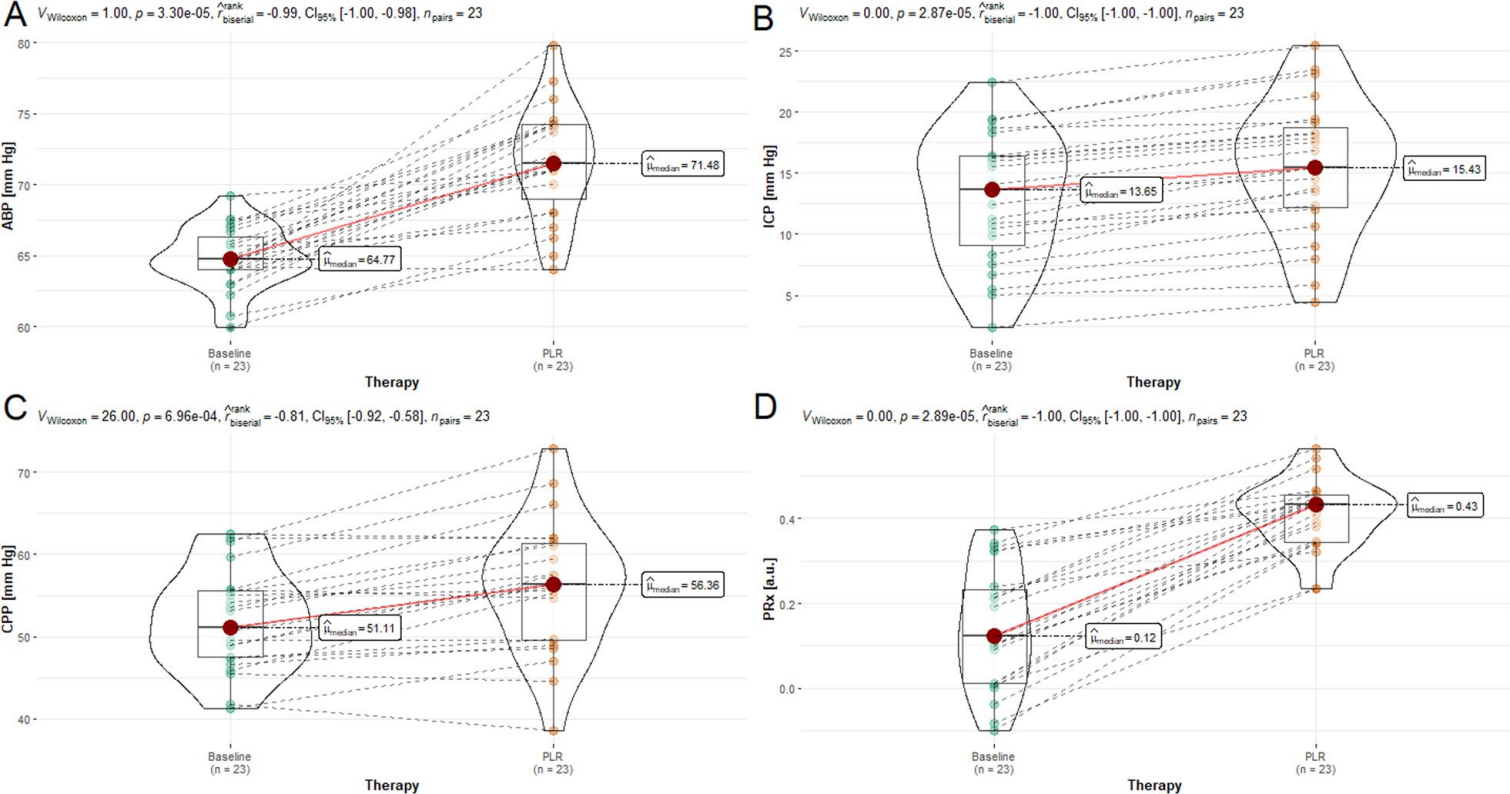
Violin plots representing the effect of passive leg raising therapy (PLR) on arterial blood pressure (ABP) (**A**), intracranial pressure (ICP) (**B**), cerebral perfusion pressure (CPP) (**C**), and cerebral autoregulation measured with pressure reactivity test (PRx) (**D**) from baseline. Values are presented as median and interquartile ranges

**Fig. 2:**
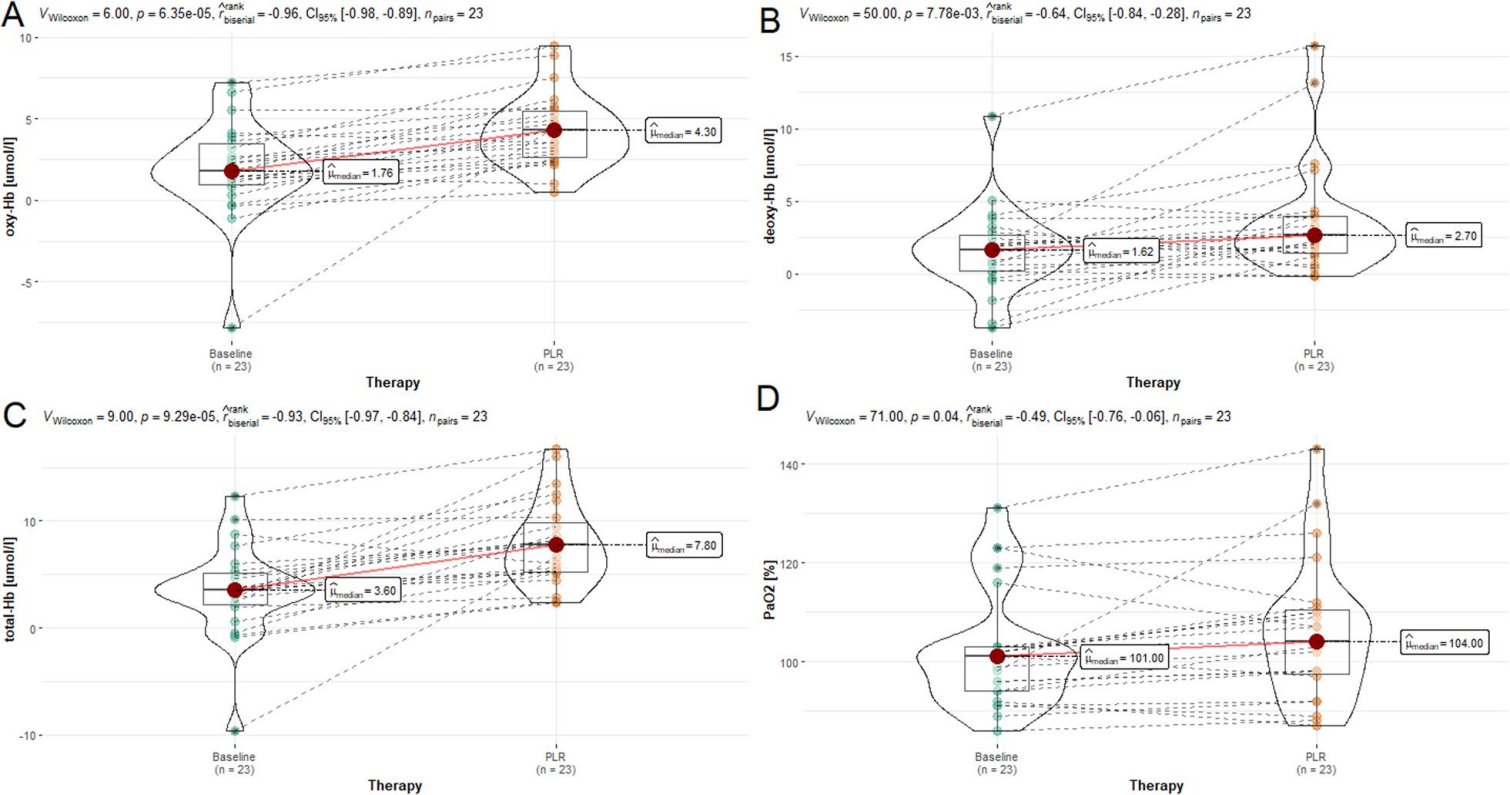
Violin plots representing the effect of passive leg raising therapy (PLR) on oxygenated haemoglobin (oxy-Hb) (**A**), deoxygenated haemoglobin (deoxy-Hb) (**B**), total haemoglobin (total-Hb) (**C**), and partial pressure of oxygen (PaO2) (**D**) from baseline. Values are presented as median and interquartile ranges

Figures 1 and 2, and Table 1 show the systemic and neuromonitoring data before and after PLR in the whole population. Considering the neuromonitoring values, PLR significantly increased ICP (*p* < 0.001), CPP (*p* < 0.001) and PRx (*p* < 0.001). Regarding NIRS-derived parameters, PLR significantly increased O_2_Hbi (*p* < 0.001]), HHbi (*p* = 0.007) and cHbi (*p* < 0.001).

**Table 1:** Hemodynamic and neuromonitoring data before and after passive leg raising

Parameter	Baseline	PLR	*p*-value	*r*_rb_
ICP (mmHg)	13.7 (8.3–16.4)	15.4 (12.0–19.2)	** < 0.001**	− 1.0
MAP (mmHg)	64.8 (64.0–66.6)	71.5 (68.0–74.2)	** < 0.001**	− 0.99
CPP (mmHg)	51.1 (47.4–55.6)	56.4 (49.6–61.5)	** < 0.001**	− 0.81
PRx (a.u.)	0.12 (0.01–0.24)	0.43 (0.34–0.46)	** < 0.001**	− 1.0
SpO_2_ (%)	99 (96–100)	99 (96–100)	0.916	0.03
rSO_2_ (%)	59 (56–68)	61 (56–63)	0.940	0.02
O_2_Hbi (umol/L)	1.8 (0.8–3.7)	4.3 (2.5–5.6)	** < 0.001**	− 0.96
HHbi (umol/L)	1.6 (0.2–2.9)	2.7 (1.4–4.0)	**0.007**	− 0.64
cHbi (umol/L)	3.6 (1.9–5.3)	7.8 (5.2–10.3)	** < 0.001**	− 0.93
PaO_2_ (%)	101 (94–103)	104 (97–111)	**0.041**	− 0.49
PaCO_2_ (%)	41 (38–43)	41 (38–43)	0.255	− 0.31

Statistically significant results are reported in bold

*PLR* passive leg raising, *ICP* intracranial pressure, *MAP* mean arterial blood pressure, *CPP* cerebral perfusion pressure, *PRx* pressure reactivity index, *SpO*_2_ systemic oxygen saturation, *rSO*_2_ regional cerebral saturation, *O2Hbi* oxygenated hemoglobin, *Hhbi* deoxygenated hemoglobin, *cHbi* total hemoglobin, *PaO*_2_ partial pressure of O_2_, *PaCO*_2_ partial pressure of CO_2_; *p*-value was obtained using paired Wilcoxon signed-rank test and the effect size was assessed using the rank-biserial correlation (r_rb_) coefficient

### Correlation between hemodynamic and neuromonitoring parameters

The Spearman correlation between neuromonitoring data and hemodynamic data is presented in Table 2. No significant relationship was found between hemodynamic and neuromonitoring parameters before PLR test. At the end of PLR test, we observed a significant correlation between MAP and CPP (*p* < 0.001), as well as PRx (*p* = 0.049).

**Table 2:** Spearman correlation coefficients between neuromonitoring-derived parameters and hemodynamic indices before and after PLR

Neuromonitoring parameters	Hemodynamic indices		
	CI	SVI	MAP
*Before PLR test*			
ICP	− 0.01	0.32	− 0.08
CPP	− 0.08	− 0.39	0.38
PRx	− 0.07	0.10	− 0.27
SpO_2_	0.03	− 0.08	0.05
rSO_2_	0.25	0.02	− 0.23
O_2_Hbi	0.04	− 0.11	0.36
Hhbi	0.15	0.24	0.08
cHbi	0.22	0.18	0.24
*After PLR test*			
ICP	− 0.09	0.21	− 0.34
CPP	0.09	− 0.23	0.70***
PRx	− 0.29	0.02	− 0.40*
SpO_2_	− 0.08	0.12	0.21
rSO_2_	0.17	− 0.25	0.06
O_2_Hbi	− 0.03	− 0.14	− 0.07
Hhbi	− 0.07	0.11	− 0.32
cHbi	− 0.01	− 0.17	− 0.38

*PLR* passive leg raising, *SVI* stroke volume index, *CI* cardiac index, *ICP* intracranial pressure, *MAP* mean arterial blood pressure, *CPP* cerebral perfusion pressure, *PRx* pressure reactivity index, *SpO*_2_ systemic oxygen saturation, *rSO*_2_ regional cerebral saturation, *O*_*2*_*Hbi* oxygenated hemoglobin, *Hhbi* deoxygenated hemoglobin, *cHbi* total hemoglobin; *p < 0.05; **p < 0.01; ***p < 0.001

### Early effects of PLR test on cerebral indexes—responders versus non-responders

Neuromonitoring variables before and after PLR therapy in responders and non-responders are reported in Table 3. In both responders and non-responders PLR significantly increased, ICP CPP and PRx. These changes were associated to a concomitant increase in the O_2_Hbi and cHbi, while HHbi increased, only in non-responders. Finally, as shown in Table 4 pall the patients showed impairment cerebral autoregulation ten minutes after PLR test; while 3 patients (13%) after the PLR and 6 patients (26%) ten minutes after PLR test showed a contemporaneous increase in the ICP and of PRx above the safety limits.

**Table 3:** Systemic and neuromonitoring data before and after passive leg raising (PLR) therapy

Parameter	ΔCI < 10%		*p*-value	r_rb_	ΔCI > = 10%		*p*-value	*r*_rb_
	Baseline	PLR			Baseline	PLR		
MAP (mmHg)	64.2 (63.0–66.7)	71.0 (66.3–71.7)	**0.004**	0.86	64.8 (64.0–66.5)	73.9 (71.0–74.4)	**0.002**	0.88
ICP (mmHg)	15.5 (11.2–18.7)	17.5 (14.5–21.3)	**0.003**	0.88	10.2 (6.2–16.1)	13.6 (8.5–18.1)	**0.002**	0.87
CPP (mmHg)	49.5 (45.4–53.5)	49.7 (47.0–59.5)	**0.032**	0.64	54.9 (48.9–60.7)	57.5 (55.7–64.0)	**0.007**	0.77
PRx [a.u.]	0.12 (0.01–0.32)	0.44 (0.35–0.47)	**0.003**	0.88	0.11 (0.01–0.23)	0.40 (0.33–0.44)	**0.002**	0.88
SPO_2_ (%)	98.5 (97.0–100.0)	98.0 (96.0–100.0)	0.540	0.19	99.0 (96.0–99.5)	99.0 (97.0–100.0)	0.656	0.13
rSO_2_ (%)	60.5 (54.0–62.0)	61.0 (56.9–61.4)	0.721	0.10	58.4 (55.8–69.0)	59.3 (56.0–66.7)	0.507	0.19
oxy-Hb (umol/L)	1.4 (0.8–2.5)	4.3 (3.0–5.3)	**0.006**	0.82	2.2 (0.5–3.8)	3.4 (2.4–5.9)	**0.004**	0.83
deoxy- Hb (umol/L)	2.4 (1.6–3.8)	2.7 (1.4–7.2)	0.328	0.29	0.44 (− 0.4–1.9)	2.5 (0.9–3.7)	**0.006**	0.79
total-Hb (umol/L)	4.6 (3.0–8.8)	8.2 (6.0–11.9)	**0.010**	0.77	3.0 (0.1–3.6)	5.5 (3.9–9.1)	**0.002**	0.88
PaO_2_ (%)	103.0 (99.0–123.0)	109.0 (102.0–126.0)	0.119	0.46	94.0 (91.0–102.0)	98.0 (90.5–108.5)	0.158	0.40
PaCO_2_ (%)	41.0 (37.0–43.0)	41.0 (38.0–44.0)	0.116	0.47	41.0 (38.5–43.0)	41.5 (38.5–42.5)	0.722	0.10
SVI (mL/m^2^)	46.0 (37.0–50.0)	41.0 (25.0–46.0)	0.083	0.52	33.5 (25.5–38.0)	41.5 (34.0–48.0)	**0.009**	0.75
CI (L/min/m^2^)	3.2 (2.9–3.7)	3.0 (2.3–3.4)	**0.019**	0.70	2.6 (2.5–3.2)	3.5 (3.0–4.2)	**0.002**	0.88
SVRI (dyn s cm^−5^ m^−1^)	2046 (1625–2396)	2121 (1888–2800)	**0.041**	0.62	2540 (2242–2711)	2147 (2004–2718)	0.272	0.31
PPV (%)	4.0 (3.0–8.0)	8.0 (2.0–12.0)	**0.041**	0.60	12.0 (7.5–13.0)	12.0 (7.5–17.5)	0.504	0.19
SVV (%)	10.0 (4.0–15.0)	10.0 (4.0–17.0)	0.959	0.01	15.0 (9.5–24.0)	15.5 (9.0–20.0)	0.784	0.07

Statistically significant results are reported in bold

*ICP* intracranial pressure, *MAP* mean arterial blood pressure, *CPP* cerebral perfusion pressure, *PRx* pressure reactivity index, *SpO*_2_ systemic oxygen saturation, *rSO*_2_ regional cerebral saturation, *oxy-Hb* oxygenated hemoglobin, *deoxy-Hb* deoxygenated hemoglobin, *total-Hb* total hemoglobin, *PaO*_2_ partial pressure of O_2_, *PaCO*_2_ partial pressure of CO_2_, *SVI* stroke volume index, *CI* cardiac index, *SVRI* systemic vascular resistance index, *PPV* pulse pressure variation, *SVV* stroke volume variation; *p*-value was obtained using paired Wilcoxon signed-rank test and the effect size was assessed using the rank-biserial correlation (*r*_rb_) coefficient

**Table 4:** Early and delayed effect of PLR on cerebral perfusion

Variables	After PLR			10 min after PLR		
	Total (*n*, %)	NR (*n*, %)	*R* (*n*, %)	Total (*n*, %)	NR (*n*, %)	*R* (*n*, %)
ICP > 20 mmHg	4 (17%)	3 (27%)	1 (8%)	6 (26%)	5 (46%)	1 (8%)
PRx > 0.3	21 (91%)	11 (100%)	10 (83%)	23 (100%)	11(100%)	12 (100%)
ICP > 20 mmHg and PRx > 0.3	3 (13%)	3 (27%)	0	6 (26%)	5 (46%)	1 (8%)

*ICP* intracranial pressure, *PRx* pressure reactivity index, *NR* non-responders, *R* responders

## Discussion

The main results of this trial can be summarized as follows: (1) despite quite preserved ICP and CPP, the PLR worsened cerebral autoregulation (PRx) and this effect was comparable in responders and non-responders; (2) an altered PRx persisted 10 min after PLR in the two groups and this was associated with a higher proportion of non-responsive patients with high ICP;(3) a positive correlation was observed between MAP and PRx, thus confirming a general loss of autoregulation in this population after PLR test.

Fluid management in ABI patients is particularly challenging, because of the changes in intravascular volume due to central neuroendocrine impairment, leading to electrolyte and osmotic disturbances [4]. A slightly positive daily fluid balance is associated with higher ICU mortality in ABI patients (odds ratio [OR] 1.10 [95% CI 1.07–1.12] per 0.1 L increase) and worse functional outcome (1.04 [1.02–1.05] per 0.1 L increase) [18], since fluid overload may directly impact on cerebral edema [32]. For all these reasons, titrating fluid balance in ABI patients is crucial and investigating the role of PLR in this population could be potentially very useful at the bedside.

However, ABI has been considered a limit for PLR use, since this maneuver could potentially worse ICP by increasing MAP and CO after the postural change of the head. In fact, head elevation at 30° in ABI patients is a standard of care [33], even if it is not strongly supported by the evidence due to lack of consistency and scarcity of data among studies investigating this topic [34]. The only study assessing the effect of PLR on ICP included only 10 patients in the acute (i.e., < 48 h) and subacute (i.e., days 5–8) phase of ABI and concluded that PLR was feasible in the acute phase since the ICP increase was self limited [35]. However, this study did not assess more advanced neuromonitoring tools, which, beside ICP, can help in the detection of altered physiology and potentially affect patients’ outcome. This is of extreme importance as altered autoregulation- defined as the ability of the brain to maintain the cerebral blood flow constant despite the systemic changes of arterial blood pressure- has been demonstrated to be associated with secondary brain damage and therefore outcome [36, 37].

Our result discourages the use of PLR in this population, as despite these were patients with stable ICP and low aggressiveness for ICP treatment**/control**, and despite ICP after PLR test increased, but not to clinically relevant values, PRx resulted importantly augmented, thus suggesting an important loss of vasomotor response and highlighting that the only use of ICP/CPP neuromonitoring may not be sufficient to detect the detrimental effects of this maneuver in ABI patients. Moreover, these effects persist at least 10 min after the end of the test. This is in line with a previous study conducted on 23 mechanically ventilated severe Coronavirus disease 2019 (COVID-19) patients, where our group demonstrated the PLR led to a reduction of cerebral autoregulatory function, assessed by using transcranial color duplex doppler technique [38]. However, PRx provides more accurate information on cerebral autoregulatory function, as compared to transcranial doppler [39].

Cerebral autoregulation is based on the functioning of a vasomotor response of intracranial vessels. If autoregulation is working, when the systemic blood pressure increases, the cerebral vessels have a vasoconstriction, which is aimed to the preservation of cerebral blood flow. This causes a reduction of ICP. As PRx is a moving and dynamic correlation coefficient between ICP and arterial blood pressure, PRx is reduced or becomes more negative [37].  If autoregulation is impaired, when arterial blood pressure increases, this does not cause cerebral vasoconstriction, but the cerebral blood flow just follows the systemic blood pressure, ICP increases and the PRx increases. In fact, in the current Guidelines for the Management of ICP, it is recommended as Tier 2 to do a MAP challenge of 10 mmHg, and assess the effect on ICP to test the autoregulation [23]

However, if MAP is beyond the upper limit of autoregulation, then the vasomotor response of the cerebral vessels is impaired, and autoregulation is altered. The lower and upper limits of autoregulation are not the same for all patients, and are likely to be individualized, according to the cerebral function. Therefore, in the post-test phase, the patients who have an increase in PRx, are those in which the upper limit of autoregulation is exceeded, whereas those in which PRx is preserved or improved are those where the MAP was still within the autoregulatory curve. Therefore, in our population the majority of patients with an increase of MAP lost autoregulatory function. Importantly, MAP may change a bit in non-responsive patients as the result of vasoactive tone increase, despite a smaller change in CO.

These results can be related to different pathophysiological mechanisms. Firstly, when the patient is put in supine position, this results in a reduced outflow through the jugular veins from the cerebral compartment, thus potentially increases venous congestion in the main intracerebral venous sinus, therefore increasing cerebral blood volume. Secondly, the PLR test may result in an increase of the intra-abdominal and intrathoracic pressures, further reducing venous cerebral outflow and potentially impairing cerebral dynamics [40–42].

In our cohort, as we included only patients with stable ICP, these effects might have had a minimal impact on CBV, but at the expenses of an impairment of the intracranial vasomotor response of cerebral vessels. This is further demonstrated by the direct correlation that we found between MAP and PRx, which suggest that when systemic arterial blood pressure increases, the cerebral blood flow passively follows MAP changes without leading to vasoconstriction but vasodilation and, hence, ICP increases and PRx becomes more positive.

Finally, rSO2 was globally mainly unchanged but HbO_2_ increased significantly both in responders and non-responders. Our results are preliminary and only few data are available so far regarding the different indexes of cerebral oxygenation [41], which must be yet validated and further explored in their efficacy to mirror physiology. rSO2 is the result of venous and arterial component, with a majority of signal related to the venous compartment [43]. This implies that rSO2 values can be related to different factors, including CPP, systemic oxygenation, venous return, cerebral autoregulatory function/metabolism and venous return potentially caused by positioning and intrathoracic pressure. We hypothesized that the increase of HbO_2_ might be mainly related to the increase of CPP, but this might not be consistent in all patients because of all the above-mentioned factors.

### Limitations

The external validity of our results is limited by the single-center design, by the number of the enrolled patients and the heterogeneity of the ABI. Worth remarking, to the best of our knowledge, this is the largest trial investigating the effect of PLR on ABI by assessing cerebral autoregulation in the context of cerebral multimodal neuromonitoring associated to continuous hemodynamic monitoring, proving data on CO. Moreover, all the patients were enrolled in a context of hemodynamic optimization and stabilization (i.e., very low vasopressor support—see Additional file 1: Table S2), when physicians usually adopt a FC to optimize fluid balance or peripheral perfusion in patients at high risk of fluid unresponsiveness, but without clinical signs of acute hemodynamic instability. Therefore, the results of this study should be considered only within safe ranges of ICP in stable ABI. Despite the heterogeneity of the population, all ABI patients have in common the fact the altered ICP and autoregulation may be detrimental in this context [44].

We stopped the data analysis 10 min after PLR assessment, so we did not evaluate more delayed effects of PLR. However, an altered PRx for 10 min should be considered as a safety limit, suggesting adopting different strategies for assessing fluid responsiveness in ABI patients. Moreover, we did not collect late hemodynamic data, which cannot be coupled to the reported late ICP and PRx changes.

The response to a FC has been assessed by the MOSTCARE™ system, whose reliability relies on the quality of the arterial pressure signal and the physicians involved in this study were highly trained in the arterial pressure waveforms quality assessment. Moreover, the least significant change (i.e., which the minimum percentage change between successive measurements considered associated with a random error and representing then a real change of CI) of this device reported in the literature is much below the threshold used to correctly identify fluid responders [45].

Despite NIRS has been widely used in this population, it has important limitations (mainly related to extracranial contamination); in addition, NIRS-derived parameters need further validations, and therefore these results should be taken with caution.

Finally, we did not estimate a sample size for this pilot study since, to the best of our knowledge, this is the first study performed in this setting by integrating neuro and hemodynamic monitoring and we could not formulate an assumption for a sample size computation based on previous data; moreover a previous investigation in this field enrolled only 10 patients [35].

## Conclusions

In ABI patient with stable ICP, PLR test increased ICP, but mostly within safety values and thresholds. Despite this, cerebral autoregulation was importantly impaired, and this persisted up to 10 min after the end of the maneuvre. Our results discourage the use of PLR test in ABI even when ICP is stable.

### Supplementary Information

**Additional file 1**. The characteristics of the patients are presented in Additional file in the Supplementary Materials 1: Table S2.

## Data Availability

The datasets used and/or analyzed during the current study are available from the corresponding author on reasonable request.

## Abbreviations

CO: Cardiac output
FC: Fluid challenge
SV: Stroke volume
ICP: Intracranial pressure
ABI: Acute brain injury
PLR: Passive leg raising
NIRS: Near infrared spectroscopy
rSO_2_: Total value of regional cerebral oxygen saturation
O_2_Hbi: Variation in the oxygenated Hb of the total rSO_2_ calculation
HHbi: Variation in the deoxygenated component of Hb within the total calculation of rSO_2_
CPP: Cerebral perfusion pressure
PRx: Pressure reactivity index
